# Discretization Provides a Conceptually Simple Tool to Build Expression Networks

**DOI:** 10.1371/journal.pone.0018634

**Published:** 2011-04-18

**Authors:** J. Keith Vass, Desmond J. Higham, Manikhandan A. V. Mudaliar, Xuerong Mao, Daniel J. Crowther

**Affiliations:** 1 Translational Medicine Research Collaboration Institute, University of Dundee, Ninewells Hospital, Dundee, United Kingdom; 2 Department of Mathematics and Statistics, University of Strathclyde, Glasgow, United Kingdom; 3 Pfizer Inc, Translational Medicine Research Collaboration Institute, University of Dundee, Ninewells Hospital, Dundee, United Kingdom; Centro de Investigación Príncipe Felipe, Spain

## Abstract

Biomarker identification, using network methods, depends on finding regular co-expression patterns; the overall connectivity is of greater importance than any single relationship. A second requirement is a simple algorithm for ranking patients on how relevant a gene-set is. For both of these requirements discretized data helps to first identify gene cliques, and then to stratify patients.

We explore a biologically intuitive discretization technique which codes genes as up- or down-regulated, with values close to the mean set as unchanged; this allows a richer description of relationships between genes than can be achieved by positive and negative correlation. We find a close agreement between our results and the template gene-interactions used to build synthetic microarray-like data by SynTReN, which synthesizes “microarray” data using known relationships which are successfully identified by our method.

We are able to split positive co-regulation into up-together and down-together and negative co-regulation is considered as directed up-down relationships. In some cases these exist in only one direction, with real data, but not with the synthetic data. We illustrate our approach using two studies on white blood cells and derived immortalized cell lines and compare the approach with standard correlation-based computations. No attempt is made to distinguish possible causal links as the search for biomarkers would be crippled by losing highly significant co-expression relationships. This contrasts with approaches like ARACNE and IRIS.

The method is illustrated with an analysis of gene-expression for energy metabolism pathways. For each discovered relationship we are able to identify the samples on which this is based in the discretized sample-gene matrix, along with a simplified view of the patterns of gene expression; this helps to dissect the gene-sample relevant to a research topic - identifying sets of co-regulated and anti-regulated genes and the samples or patients in which this relationship occurs.

## Introduction

The prevalent reductionist and historically successful approach to biology has largely depended on analytical methods focusing on single genes or proteins to infer interaction partners. In many model systems the paradigm has been to perturb or mutate a single gene and observe what happens; pull-down or yeast two-hybrid experiments have the same aim, connecting target proteins to those which they bind to, while many *in vitro* studies have shown that perturbation of a single gene is usually associated with concerted changes in many genes. Numerical methods have attempted to look for larger groups of genes which are inferred to be co-regulated using “guilt-by-association” arguments [1]. A more ambitious approach has been to use observational microarray experiments to infer which genes are driving the observed expression patterns [2]–[4].

We suggest that in a group of unrelated individuals multiple polymorphisms are one cause of modulation of the expression of many genes, dramatically extending the single gene-perturbation paradigm. Consequentially, most expressed genes in any tissue will either be directly affected by polymorphisms or will be perturbed by the primary affected genes. Additional causes of expression perturbation include the presence or absence of alternative haplotypes, operating in *cis* or *trans*, to affect transcription [5], [6]; copy number variation reflected in the abundance of transcripts [7]; in cancer studies, mutations, loss-of-heterozygozity [8], gene-translocations [9], amplification [10] and epigenetic effects [11] all add to the natural genetic heterogeneity. Furthermore it is likely that microRNAs will display the same variability as other biological molecules, giving rise to concerted abundance changes [12]. In addition to genome differences, microarrays of normal lymphocytes from randomly selected subjects reveal effects due to time of collection, age, sex and nutrition [13]. The end result of this heterogeneity is that gene-expression is substantially different in every individual, regardless of disease; despite this, a single tissue maintains a recognizable phenotype; the “system” state is regulated, so we expect the same control processes to be used in many samples. Consequently we would expect many changes to be correlated in large studies of unrelated individuals. If this argument is correct, it predicts that many relationships would occur repeatedly, far more often than would be expected by chance. This argument is consistent with the idea of bistability, revealed by network analysis, predicting sets of genes that are co-ordinately up- or down-regulated [14].

It is the aim of most microarray studies to identify patterns of expression, common to several samples [15], [16]. If we restrict ourselves to examining relationships which have passed some “relevance” test and ignore details of what is happening in individual subjects or patients we simplify the analysis and increase the opportunities to discover large-scale patterns. We set out to find if correlation between gene transcripts exceeds expectation and if this information can identify known and plausible new transcriptional relationships. A less-easily evaluated goal is to identify sets of genes which are strongly co-expressed, but without any obvious shared control; these can either be identified from global patterns or by studying a targeted subset which share some biological role. This approach has been discussed recently by Quigley and Balmain who attempt to use expression correlation-networks to augment genome–wide association studies (GWAS) and used this methodology to compare human-cancer with mouse genetic studies [17]; the stratification of patients, suggested from this approach, is simplified by our discretized-data.

We describe an unsupervised network construction method, based on analyzing the relative frequency of co-expression of two genes following discretization. Unlike Chuang et al [18], who use prior pathway knowledge to examine the plausibility of a pathway's involvement, all assessment of biological interpretation in our methods is retrospective; we first construct a network, then examine its structure to identify highly-connected sub-graphs and take these groups of genes to look for common biological roles. Analyses generating unsupervised networks allows an objective assessment of how improbable their size is, free of prejudice on what the relevant pathways are, or indeed if they have been identified. We discuss the relative merits of this approach with a standard correlation analysis.

Validation of networks with biological results is a difficult area and this has been attempted, to some extent, by simulating microarray-like data using some form of numerical modelling [19], [20], but it is generally accepted that “assessments of methods performances remains a challenge… systematic validation is crucial, since it shows strengths and weakness of the methods” [19]. We attempt to identify potential co-expressed genes, from the definitions, used to define the model used by SynTReN [20] to build their synthetic data and to compare these with our calculated networks. Most of the expected relationships in this system are due to indirect effects, that is path-lengths of 2 or more. It is important to consider these transitive relationships as they explain many of the inferred co-expressions; these would otherwise be considered as false-positives, although they are expected consequences of the relationships used to model the system.

Many network identification methods have been proposed [3], [21], [22] with the aim of finding causal relationships; our approach has a different aim, to identify co-expression-cliques based on a simple discretization table; this table is not merely a step in the algorithm but can be subsequently revisited to reveal the samples in which a gene-cluster is switched on, to associate patterns discovered from network analysis with relevant subjects. From a clique we expect to find some samples with most of the genes uniformly switched off or on and this is easily revealed by summing the discretized values for all these probe-sets.

## Results

### Practical considerations used in building and evaluating gene-expression networks

We simplify microarray analysis by converting the real values in raw data into 3 discrete values: −1 is down, 0 is around the mean value and +1 is up (see Experimental Procedures). This simple concept summarizes many biologists' informal view of gene-expression, often discussing only direction of change – up or down. This gives a discretized matrix, from which we calculate three possible relationships: ***mm*** (minus:minus, down-together), ***pp*** (plus:plus, up-together) and ***pm*** (plus:minus, up-down). These relationships can be formatted as pair-lists: ***pp***, ***mm*** and ***pm*** to compare networks from different datasets; in the case of ***pm*** gene1 is up and gene2 is down. Gene pair-lists are crucial to the practical network interrogation; to identify co-regulation for a signaling pathway its genes are first arranged in all pair-wise combinations, which are then used to detect observed gene-pairs from a set of biological samples.

Three datasets are used in this study: first, the San Antonio Family Heart Study (SAFHS) [23] has produced genome-wide transcriptional profiles of lymphocyte samples from 1,240 participants; second, 166 subjects from mixed European- and Asian-derived populations by Cheung and Spielman [24] were used to establish Epstein Barr virus immortalised lymphoblastoid cells which were grown in cell-culture and the transcripts then analysed; third Decode study GSE7965 with peripheral blood samples from 1021 subjects [25]. SAFHS used Illumina chips while Cheung and Spielman used Affymetrix Focus chips. We compare networks from these two datasets and the large size of the SAFHS allowed us to subdivide it into two independent subsets of 620 individuals. The use of different microarray technology between Cheung and Spielman data and SAFHS further reduces the possibility of technical artefacts and emphasises the wide applicability of our methodology.

### Validating the identification of correct relations

**Figure 1:**
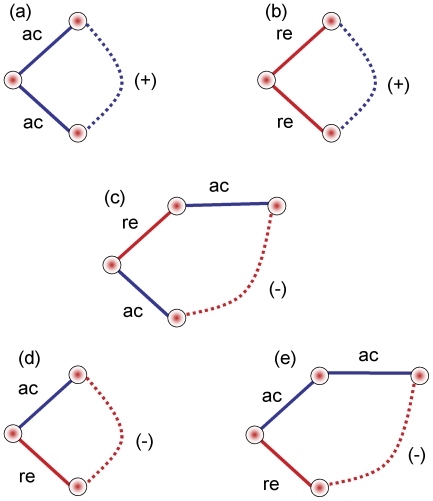
Predicted transitive relations in a SynTReN model network. The definitions used by SynTReN to model synthetic data **ac** (positive-regulation) and **re** (repression) are illustrated with the effector on the left. The targets with transitive relations, either positive or negative are shown connected with a dotted edge. Five simple motifs are illustrated, but scope for more complexity exists when these relationships overlap. Positive co-expression is predicted by either **ac** or **re** definitions, but the two targets have to be connected to the same effector by the same relationship for this to be true (**a** & **b**). Negative co-expression needs some form of asymmetry, as shown in **c–e**. The success of our predictions depends on how the simulation is set up; we used 100 genes with known relations and 100 background genes, in the comparisons shown in **Table 1**, but decreasing the number of background genes increases the complexity of the expected transitive relationships.

**Figure 2:**
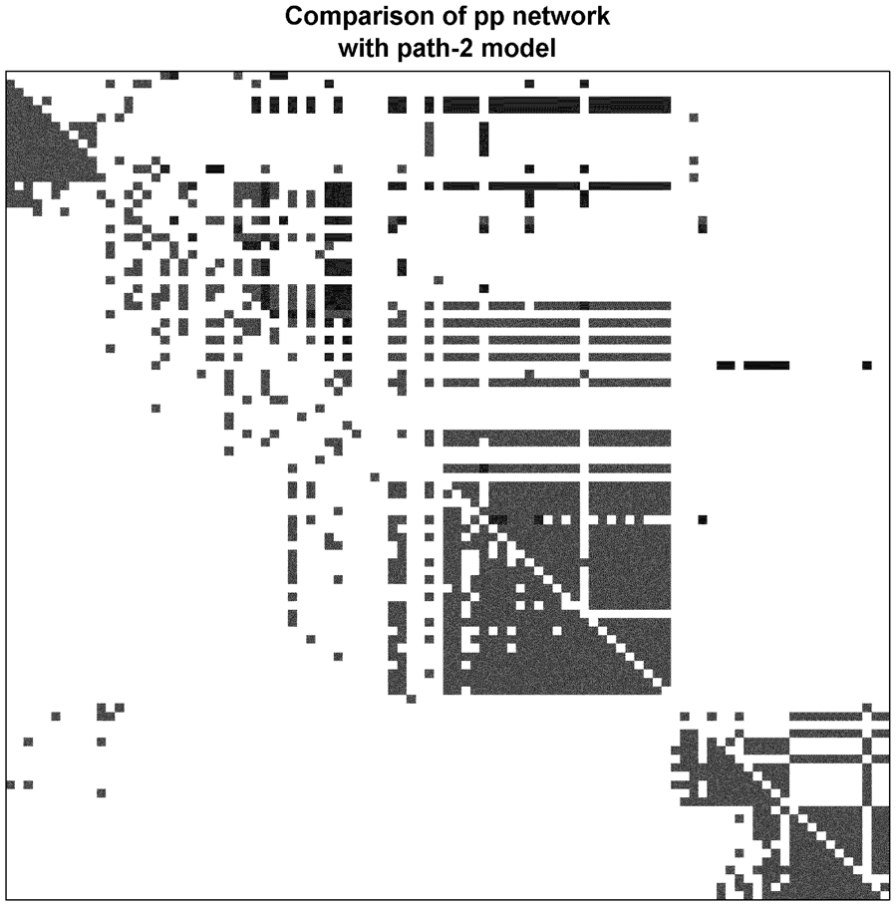
Comparison of pp identified gene-pairs with transitive path-length 2 pairs from *E coli* transcriptional definitions. An adjacency matrix was constructed, where the *E coli* definitions ***ac*** was set to 1 and ***re*** set to −1; ***du*** relationships were set to 0 and are therefore ignored in this analysis. This adjacency matrix, **A**, was squared (**A.A**) which reveals paths of length 2; in this qualitative analysis no allowance is made for loss of relationships due to positive and negative values summing to zero. This *E coli* definition derived matrix is the upper-triangle in the diagram and the gray squares are positive and black are negative. The lower-triangle is the ***pp*** matrix calculated from the SynTReN simulated data for 100 samples.

The simulation package SynTReN [20] builds microarray-like data files based on a set of known transcriptional interactions (between *E coli* genes in our test). Synthetic data from this program have been used to validate network discovery methods for microarrays [26]. Comparisons between the relationships used to define the SynTReN data generation and the recovered networks have been numeric, without apparent consideration of the type of interaction. We have used a network approach to infer transitive relations for positive gene-interactions to estimate sensitivity and, more tentatively, specificity of our approach. Three interactions are defined for *E coli*: ***ac*** (positive interaction), ***du*** (dual-action) and ***re*** (repressor). Two of these are consistent with our defined interactions: ***ac*** is equivalent to ***pp*** or ***mm***, while ***re*** is our ***pm***. We do not infer a causal link from our relations. If we simply ask for our relations to detect the original definitions, then our method does well, correctly identifying between 70 and 95% of the correct type of relationships and less than 10% of the incorrect (**Table 1**). However specificity is less certain as the original gene-definitions account for less than 10% of the edges in our predicted networks. This comparison does not take into account transitive relations (**Figure 1**); if gene1 is connected to gene2 by ***ac***; and gene1 to gene3 by ***ac***; this implies that gene2 and gene3 will also have a positive relationship (**Figure 1a**); these are readily calculated by using the ***ac*** definitions to build an adjacency matrix, squaring this gives the nodes (genes) connected by path-length 2. **Figure 1 (b–e)** shows other expected transitive relationships. As this analysis is only relevant for ***ac*** (our ***pp*** and ***mm***), we now have an extended target which matches between 30 and 80% of the number of gene-pairs in our analysis. About 75% of, ***ac*** defined, path-length 2 pairs are found in our ***mm*** and ***pp*** networks, but only 2% or fewer in the ***pm*** pairs. Significantly these calculated path-length 2 connected-pairs, together with the direct ***ac*** definitions, now account for between about 30 and 67% of all our ***mm*** and ***pp*** edges. If we sum path lengths 1–3 relationships, for cluster 4 (**Figure 2**), the predicted and observed are in approximate agreement. As we do not claim that this approach predicts all expected gene-pairs, this seems a good validation of the detected ***pp*** and ***mm*** using the SynTReN system, but a formal identification of all expected relationships, even in this synthetic system, is impossible as several conflicting definitions are used to build the model for the data synthesis. In **Figure 2**, we have supplemented this analysis by including ***re*** as −1 and ***ac*** as +1 in an adjacency matrix to calculate possible transitive relationships and compare this to the calculated ***pp*** network, from two independent SynTReN generations of 200 samples; only common gene-pairs are accepted. The two networks, generated in these fundamentally different ways, are very similar. It is clear that the path-lengths of greater than 2 can explain why the observed networks have more highly connected sub-graphs for two clusters (c2 and c3).

**Table 1:** Estimation of consistent identification of the *E coli* transcriptional classification.

	*ac*Σ97	*du*Σ11	*re*Σ 41	*ac* path length 2Σ = 837	*ac* & *re* *+1 −1*	
100 samples*pp* (Σ = 1082)*mm* (Σ = 1054)*pm* (Σ = 1169)	80 (82%)89 (92%)0	454	2329 (71%)	575 (69%)620 (74%)0	71575038	2320316
200 samples*pp* (Σ = 1668)*mm* (Σ = 1373)*pm* (Σ = 2152)	88 (90%)92 (95%)0	556	2233 (89%)	638 (76%)653 (78%)10 (1%)	84183740	2523345
300 samples*pp* (Σ = 1972)*mm* (Σ = 1605)pm (Σ = 2870)	93 (96%)92 (95%)0	556	3336 (87%)	652 (78%)667 (80%)12 (1%)	87186443	2925361
400 samples*pp* (Σ = 2315)*mm* (Σ = 1938)*pm* (Σ = 3605)	93 (96%)93 (96%)0	556	3336 (87%)	662 (79%)671 (80%)15 (2%)	88387344	2826368
2×200*pp* (Σ = 1253)*mm* (Σ = 1114)*pm* (Σ = 2172)	91 (95%)87 (90%)0	556	3332 (78%)	630 (75%)640 (76%)6 (1%)	85780141	2821337

We assess the correctness of our identified gene-pairs with the E coli activation (***ac***) and repression (***re***) relationships used by SynTReN to build the networks. This is equivalent to a check on specificity. We additionally wished to identify gene-pairs which were highly likely to occur, based on the ***ac*** definitions, but including transitive relations, that is - all the genes that are connected by an ***ac*** network path-length of 2. This 2-path network is not a full prediction of all observed relations in the data-file as it does not include the ***du*** and ***re*** pairs. We calculated the sum of the *E coli* definition adjacency matrices for ***ac*** (+1) and ***re*** (−1) for path-length 1, 2 and 3 and again compared this network with our identified pairs. The results with correlation analysis are almost the same as those found by discretization.

The detected network size increases along with number of samples (**Table 1**) suggesting that using larger numbers of samples infers more and more relationships or is now detecting noise, most of which we cannot predict from the *E coli* gene-definitions. This suggests that best practice for the method is to use some form of random sampling followed by selecting only pairs that occur in more than one sampling (**Table 2**) as this should specifically remove pairs due to low variance genes, which have no *E coli* definitions and are presumably only affected in the SynTReN simulation by added noise. This argument depends on random numbers being generated with different values during each simulation, This appears not to be the case (see below).

**Table 2:** The use of independent studies to increase specificity in network determination.

			*ac*	*du*	*re*	Low-variance pairs
Subset 1	***pp*** ***mm*** ***pm***	154713432083	92900	556	3333	14128
Subset 2	***pp*** ***mm*** ***pm***	162113912099	92890	556	3334	9105
Subset 1 AND 2	***pp*** ***mm*** ***pm***	125311141364	91870	556	3332	450

SynTReN was used to build a synthetic dataset of 400 samples, these were randomly subdivided into two subsets of 200 each. The discretization-based co-expression networks were calculated for each and the shared edges used to give a third network. The 10% of the genes with the lowest variance were selected and the possible gene-pairs for those determined, all of these genes were not defined by **ac**, **du** or **re** relationships. The low-variance based gene-pairs detected are preferentially discarded by this procedure, suggesting that this is one reasonable technique for discarding false relationships.

### Estimation of false relations

We detect some ***du*** and ***re*** pairs in our ***pp*** and ***mm*** networks; however, these are mostly defined as ***ac***-connected by paths of length 2, so are correct ***pp*** and ***mm*** pairs by this criterion. This argument suggests that almost all the apparently false E coli definitions are in fact correct. This still leaves the extra relationships, not defined by the *E coli* model to explain. If we assume that “correct” or true relationships are those that are directly or indirectly (transitive relationships), defined by the *E coli* transcription model, used in the SynTReN simulation, we can count the network relations which do not fit this criterion. However this is likely to overestimate the “incorrect” pairs as we find about 15% of the pairs in ***mm*** and ***pp*** relations come from this group but less than 2% in ***pm*** networks. It is likely that these undefined genes form relationships by their relative invariance in the model as noted in the original SynTReN paper [20]. In a simulation data file, if genes with less than half of the modal variance were selected, 77 out of 78 genes are undefined. Almost all these are designated as background genes by SynTReN and given the prefix “***bgr_***”. It is difficult to set an optimal cut-off for excluding genes by low variance with the SynTReN data and this is likely to be much more of a problem with real data. Our inability to exclude all the ***bgr_*** genes may not be a failure of our algorithm as comparison of ***bgr_*** containing gene-pairs between two runs of SynTReN shows an extremely non-random result. The correlation between the ***r*** values, linking these genes, between two the runs is 0.98, so it appears that background modelling in SynTReN is non-random. This also explains our inability to exclude FALSE relationships by comparing multiple simulated data-sets. We find that these genes contribute about 15% of our co-expressed relationships, consistent with this relative invariance. However the undefined genes do not appear in any cluster identified by spectral analysis [27] in our ***mm***, ***pp*** or ***pm*** networks (not shown), so their inclusion in the network does not interfere with our aim to identify significant patterns of co-expression, which we believe is an essential prerequisite to discover reliable patterns in our networks. The ***pm*** analysis has not been formally analysed in the same way, but it appears that a combination of transitive ***ac*** relationships, together with a small number of negative ***re*** relationships do account for a substantial number of the relationships found. It is clear from **Figure 2** that clusters **c1** and **c3** are expected to have a ***pm*** relationship, from the ***re*** path-length 2 links between the two; in fact ***pm*** relationships between the 3 clusters in the ***pp*** network account for about 50% of all the ***pm*** pairs.

We have compared our approach with those methods summarized by Pihur *et al* [26] and they show the same effect of increasing the ***fdr*** as we find by using larger number of samples – the number of detected edges increase. In their case they are lowering their confidence limit, in ours, using larger number of samples paradoxically increases the number of edges which we cannot justify theoretically. This suggests that less plausible edges are being added, our structural approach to the “correct” theoretical network supports this. Most path-lengths 1 and 2 relationships are detected early and it is clear that longer paths explain the “filling out” of clusters **c2** and **c3** (**Figure 2**).

### Consistently Detected Relationships

The SynTReN simulated data for 400 samples was randomly partitioned into 2 equal matrices and the network analysis carried out on both subsets. Around 20% of the edges were discarded when the two subsets were compared, however using the *E coli* definitions as our standard only 1 or 2 of these relationships are lost, these includes the ***du*** and ***re*** relations detected by the ***pp*** and ***mm*** pairs. All these networks were also compared to the genes with lowest variance in the simulated data, which we suggest as candidates for incorrect relationships, in the ***pp*** and ***mm*** pairs these were substantially reduced in the networks identified by the intersection of the 2 subsets (50–65%) and completely lost with the ***pm*** intersection (**Table 2**).

### Effect of Noise

The activating (***ac***), dual (***du***) and repressive (***re***) relationships give one measure of correct inference by our program or a reverse-engineering approach, such as Aracne. Using this criterion both do very well and only begin to fail to detect TRUE pairs at high noise levels (bio-noise of 0.35–0.5), data not shown; however both programs are strongly affected by noise and identify many relationships that only appear to be affected by noise. SynTReN conveniently prefixs these genes with “**bgr_**”; as well as these FALSE relationships many additional gene-pairs are detected at high noise levels, whose connections must be considered as dubious. It is possible to filter out many of these FALSE and suspect pairs at lower noise levels (0.1–0.3) by excluding genes of low variance; this does not work at higher noise levels. Both our program and ARACNE can reduce this problem by decreasing the probability cut-off used, however this has the undesirable effect of losing TRUE relationships. While we were developing our discretization approach we were also using correlation to determine probable positive and negative relationships and were aware of very great similarities in the resulting networks – the main difference was that discretization could subdivide both positive and negative correlations into ***pp***, ***mm***, ***pm*** and ***mp*** pairs. We used positive and negative correlation identified pairs to try to filter out FALSE gene-pairs; correlation analysis with a value of ***r*** as low as 0.1, equivalent to a p-value for two-tailed testing of 0.32, removes around 80% of pairs containing “bgr_” in both low- and high-noise cases. This also holds for Aracne. When noise is high this filtering loses from 10 to 25% of the TRUE relationships at the highest value of ***r*** tested (0.3, equivalent to p-value of 0.0024); the signal to noise improves dramatically. As we do not have a definite number of TRUE relationships we choose to compare our known repressive and activation definitions with the “bgr_” pairs, this information is shown in Table 3. Two main conclusions come from these results – first, the correlation filter removes very few of the TRUE relations, even when filtering at the highest ***r*** value; second, the bgr_ pairs are significantly removed, even at ***r*** of 0.1. Although correlation has weak interpretative power, compared with discretization, it offers a powerful improvement to the method and carries the benefit of well-understood probability inference. The remaining “bgr_” pairs do not pose a problem to identifying cliques of co-regulated regulated genes, as we aim to do, for biomarker identification; the “bgr_” pairs are poorly connected and are clearly separated, by eigen- or SVD-reordering, from genes involved in real modelled simulation.

**Table 3:** Effectiveness of correlation network as a filter.

Bio-noise	*r* for filter	Network	*ac*	*re*	*bgr_*
**0.1**	**0**	***pm***	4	36	5707
		***pp***	94	7	3095
		***mm***	94	8	1758
	**0.1**	***pm***	1	36	1240
		***pp***	90	7	632
		***mm***	91	8	410
	**0.3**	***pm***	1	36	20
		***pp***	90	4	16
		***mm***	91	4	11
**0.5**	**0**	***pm***	12	37	15101
		***pp***	91	13	7213
		***mm***	91	13	7148
	**0.1**	***pm***	1	35	3086
		***pp***	89	3	1394
		***mm***	90	3	1466
	**0.3**	***pm***	0	27	141
		***pp***	84	3	87
		***mm***	84	3	86

The discretization analysis was performed at two levels of “bio-noise” 0.1 and 0.5. Positive correlation was used as a filter to remove edges not present by correlation from ***pp*** and ***mm*** networks. Negative correlation at the three ***r*** levels was required for ***pm*** edges to be retained. With 0.1 noise, correlation removes almost no TRUE edges while removing most of the FALSE (bgr_) pairs.

Analysing real observational data is less clear as noise is unknown and many samples may not show a relationship important for other individuals – in cancer studies an activated oncogene may condition the controls operating in a subset of patients. Our analytical settings, based on SynTReN simulations, must therefore only be considered as guidelines for real data, but show that it is easy to greatly improve network inference by this simple technique.

### Validating biological relationships in the networks

Any new method should detect previously identified information which we can generate using published analyses. We used a set of genes identified in the Cheung and Spielman [20] data as differentially expressed between European and Asian subjects. These were divided into two groups European-up (**Eu**) and European-down (**Ed**) and these separate lists used to build gene-pair lists, **Eu : Ed**. The gene-pair list has all possible combinations of the genes in **Eu**, in column 1, with the genes in **Ed**, in column 2; we expect the relationships to be in the opposite sense in Asian subjects. While we expect these relationships to occur commonly in the data we do not expect all genes to be uniformly up-regulated in one population or down in the other and in the original paper there is a spread of variances to support this view. In our analysis we would expect to find these pairs predominantly in our ***pm*** or in the negatively correlated networks as they would be predicted to behave in the opposite way in both European and Asian subjects. We looked for the 258,096 possible **Eu : Ed** pairs in networks built by discretization (Z = 0.4) and by correlation at nominally similar significance cut-off (P<0.005). Only the Cheung and Spielman data revealed significant differences between the expected match in the ***pm*** network (60%) and ***pp*** or ***mm*** (2%); similar results are found using correlation (**Table 4**). With discretization ***mm*** and ***pp*** (negative control) only 2.5% of the pairs were found but ***pm*** matched 55%; correlation (correlation coefficient = 0.29, n = 166) did less well with positive correlation matching 1.7% and negative correlation only 25%. These comparisons show that the expected gene-pairs, from the published data, are found with reasonable sensitivity, 60% or 39% for discretization and correlation respectively. The lack of matches to the ***mm*** and ***pp*** pair lists, 2%, shows that good specificity within the same dataset is found by both methods. When matches to the same **Eu : Ed** gene pair-list are looked for in the SAFHS or Decode the specificity is lost (**Table 4**), this is the expected result, as the original patterns were due to differences between the European and Asian subjects in the Cheung and Spielman data, which would not be expected to be found within the Mexican-American or Icelandic populations.

**Table 4:** Assessment of predicted *pm* relationships from European versus Chinese and Japanese data.

	*pp*	*mm*	*pm*	+ve corr	−ve corr
Cheung	5 053 (2%)	4 911 (2%)	155 326 (60%)	5 880 (2%)	101 862 (39%)
SAFS	36 342 (14%)	40 268 (16%)	45 439 (18%)	40 248 (14%)	42 054 (16%)
Decode (all)	33 921 (13%)	34 831 (14%)	47 699 (18%)	46 272 (18%)	48 574 (19%)
Decode (male)	3 601 (1%)	3 595 (1%)	5 397 (2%)	48 950 (19%)	51 212 (20%)
Decode (female)	5 980 (2%)	5 952 (2%)	9 010 (3%)	38 034 (15%)	39 730 (15%)

Genes with significantly different expression between Asian and European subjects were identified by Spielman et al [20] and we divided these into two groups - European-up (**Eu**) and European-down (**Ed**), using the average expression for Europeans minus the average expression for Asian (Chinese and Japanese). These two probe-lists were used to make a pair-list of all possible combinations of **Eu : Ed**, and filtered to only contain the probes which appear in our final discretized data (Z = 0.4). For comparisons with the non-Affymetrix data (SAFHS and Decode) this Affymetrix probe pair-list was converted into a gene symbol pair-list. The comparisons show the number of common unique pairs between the networks and the **Eu : Ed** pair-list.

### Numerical assessment of networks

The reproducibility of networks identified by discretization was examined by comparing the gene-pair lists for two randomly selected subsets of SAFHS. The Z-score cut-off was set at a low value (Z = 0.4), allowing small, but detectable, changes in gene-expression to be evaluated; as a result the networks contain many edges. The ***mm*** and ***pp*** networks were found to share many edges (10×10^6^, 66%) both within and between the randomly-selected subsets, see **Table 5**; genes which are down-together are also often up-together. The ***pm*** networks from the subsets showed a similar level of shared edges (23×10^6^, 72%) (**Table 5**). However comparing ***mm*** or ***pp*** to ***pm*** finds almost no shared edges (1×10^3^, 0.001%), suggesting a highly specific exclusion of ***pm*** relationships from ***mm*** or ***pp*** even in independent sample subsets.

**Table 5:** Comparison of discretized networks from 2 subsets of SAFHS subjects.

Comparison of 2 randomly selected independent subsets of SAFHS(620 and 619 subjects) (edges ×10^3^)					
	mmB	pmA	pmB	ppA	ppB
**mmA (15523)**	**10787**	0.1	1.4	**9864**	**9656**
**mmB (16548)**		1.2	0.01	**9819**	**10483**
**pmA (21093)**			**14400**	0.045	1.0
**pmB (22119)**				0.8	0.003
**ppA (16071)**					**10653**
**ppB (16723)**					

“Duplicate” information is discarded in these comparisons; reasons for duplication include multiple probesets for single genes and in the ***pm*** networks relationships going in both directions. Networks were constructed by the discretizion (Z = 0.4) or correlation methods from two randomly selected sample subsets of the SAFHS dataset. The number of edges in each of the networks is given in brackets (×10^3^).

It could be supposed that what we are seeing in our networks is strongly influenced by “noise”. In an attempt to address this, the gene-sample discretized matrix was randomized and the network calculation repeated, to estimate the number of edges or network ***size*** we expect by chance association. Randomization gives networks of only 5% of the normal size, with Z = 0.4 (**Table 6**); when the cut-off is increased to Z = 1.6, the randomized data gave a network size of about 1% of the normal size (data not shown). As a further test, multiple randomization runs (n = 100) were used to estimate the probability that observed network size could be due to chance; using the t-test to assess the chance of constructing a network of the observed size, due to random effects, finds P of approximately zero (t = −10730.284 for Z = 1.6 and t = −72926.2102 for Z = 0.4). Edge-by-edge comparison of true with randomized networks is very revealing; when ***mm*** or ***pp*** networks were compared to their randomized counterparts the number of *shared* edges is about 1% of the true network size. When ***mm*** or ***pp*** networks were compared to the randomized ***pm*** networks the number of edges in common is now about 2-fold higher, showing that the normal lack of shared edges is lost (**Table 6**). The extremely low coincidence of edges between ***pm*** and (***mm*** or ***pp***) implies that the networks do contain very specific information; this is reinforced by the same comparison between two sample subsets (**Table 5**). We find however that comparing common ***mm*** and ***pp*** pairs between subsets A and B does not give an enhanced intersection, perhaps giving extra credence to the two network types carrying different information. This is not true in SynTReN modelled data where we find structures indicating symmetry.

**Table 6:** Discretized networks carry consistent information.

Effect of randomization on specific information in networks(Cheung and Spielman, Z = 0.4) (edges ×10^3^)					
	pm	pp	Randomizedmm(80)	Randomizedpm(79)	Randomizedpp(159)
**mm (2177)**	18	1466	15	30	15
**pm (2875)**		18	21	40	21
**pp (2180)**			15	30	15

Networks were constructed from discretized (Z = 0.4) data for all the Cheung and Spielman subjects, with the total number of edges shown in brackets. The left-hand 2 columns show the number of shared edges for un-shuffled discretized gene-sample data, while the right-hand 3 columns give the result of the comparison between the un-shuffled and shuffled gene-sample networks. Randomization was carried out for each row of the gene-sample discretized table using the R-package function “sample”.

### The networks are robust to different microarray technologies

These two studies differed in two important respects: first, they used different microarray technologies and second, the SAFHS study directly measured the RNA from isolated cells [23], while Cheung and Spielman used immortalized lymphoblastoid cells, subsequently grown in tissue culture to minimize environmental effects [24]. For all these reasons we expect the shared patterns of gene-expression to be low but we looked for any specificity indicating that the technique could pick out meaningful shared biological patterns. Network comparison within single datasets showed high specificity: within the Cheung and Spielman data like the SAFHS ***mm*** and ***pp*** networks share many edges, while the ***pm*** network has few shared edges with either ***mm*** or ***pp*** (**Table 7**). When the Cheung and Spielman ***mm***, ***pp*** and ***pm*** are each compared with all three (***mm***, ***pp*** and ***pm***) networks from SAFHS they always find more pairs in common with the homologous networks. So it is clear that specific effects are found despite the biological and technical differences between immortalized lymphoblastoid cells, grown in tissue culture, and assayed with Illumina microarrays and white blood cells directly isolated from blood and analyzed using Affymetrix Focus Genechips®.

**Table 7:** Discretized networks carry consistent information.

Comparison of networks from Cheung and Spielman (C) and SAFHS (S) (×10^3^)					
	*pm*C	*pp*C	*mm*S	*pm*S	*pp*S
***mm*** **C (2177)**	18	1466	**482**	**297**	**448**
***pm*** **C (2875)**		18	**386**	**614**	**349**
***pp*** **C (2180)**			**464**	**277**	**432**
***mm*** **S (23368)**				872	16697
***pm*** **S (24571)**					848
***pp*** **S (31006)**					

The networks were derived from discretized data (Z = 0.4) for both the SAFHS (S) and the Cheung and Spielman (C). For comparison purposes the platform specific identifiers were converted to gene-names and any resulting probe-set redundancy eliminated. Only the gene-names represented on both the Illumina and Affymetrix chips were used in this comparison. The numbers for comparisons between the different datasets are shown in bold.

### Does discretization have any advantage over correlation?

If the discretization approach is to have any merit, over correlation, it should identify asymmetrical relationships. The connectivity of two transcription factors *RUNX1* and *RUNX3* changes dramatically in the SAFHS ***pm*** (Z = 0.4) network; when they are up-regulated *RUNX1* is connected to 927 genes and RUNX3 to 1584, when down *RUNX1* has 3874 partners and *RUNX3* only 988. The RUNX genes are known to be important in normal haemopoiesis [28] and *RUNX3* has been found to suppress CD4 in T-cell differentiation [29], which we also observe here as a ***pm*** relationship in the SAFHS, along with the suggested links between *RUNX3* and the proteolytic enzymes granzyme and perforin found in effector T-cells (not shown).

Identifying gene-pairs with symmetrical behaviour, however, does give insight into network structure. We evaluated the reciprocal nature of the relationships by counting shared edges in ***mm*** and ***pp*** networks, from separate, randomly-selected sample subsets (**Tables 5**). Over 60% of the edges were shared between the networks, suggesting that it is common to find the same pair of genes both up and down-regulated together. The gene-pairs that passed this test are more highly conserved between the two sample subsets with the overlap between the ***mm:pp*** intersections being around 73%, about 10% higher than for the single ***mm*** or ***pp*** networks (not shown). In ***pm*** networks, also about 60% of the gene-pairs exist in both up-down senses (***pm:mp***); here again a slightly higher level, about 70%, were found between these reciprocal-pair networks from the two sample subsets, so the up-down pairs, which are found in both senses, are found more reproducibly (data not shown). Not one common pair existed between the ***mm:pp*** and ***pm:mp*** intersection networks, even between the separate subsets, where random effects might have been predicted to give some common pairs. This dramatically illustrates the specificity of the determined relationships and implies that the networks have a consistent structure. We do not propose this is a good method for discarding “unreliable” relationships as we believe that many crucial control effects will be asymmetric.

Using the SAFHS data, we compared the networks derived using our discretized data followed by selection against a Monte Carlo calculated cut-off with correlation analysis. For the correlation-coefficient cut-off of +0.1032 about 60% of the edges are also found in our ***mm*** and ***pp*** networks (Z = 0.4), but only 2% in common with the ***pm*** networks. This result is reversed with correlations of less than −0.1032, with ***mm*** and ***pp*** only matching 2% of the edges but ***pm*** now sharing about 65% of the edges, see **Table 8**, showing that the relationships identified by the two methods are consistent.

**Table 8:** Discretized and correlation networks share many relationships.

Comparison of discretization and correlation networks (edges ×10^3^)						
	Correlation>0.1032(12900)			Correlation<−0.1032(20000)		
Discretization networks	Only in discretized	Both	Only in correlation	Only in discretized	Both	Only in correlation
***mm*** ** (10300)**	2600	7600	5300	1000	300	19700
***pp*** ** (10300)**	2500	8800	4100	1000	300	19700
***pm*** ** (12800)**	12500	350	12500	4500	8300	4500

Tabular Venn-diagrams show the shared information between networks constructed using discretization and correlation methods; both methods were applied to the two subsets of the SAFHS. The networks from each subset, for each method, were compared and only the gene-pairs found in both subsets were used for the comparison. The comparison between discretized and correlation networks is described in Methods. All duplicate gene-pairs, resulting from multiple probes, were eliminated – leaving only one gene-pair for each relationship; here the direction of the ***pm*** relations is ignored. The size of each resulting network is included, in brackets.

### Identification of a bi-phasic network for central metabolic pathways

**Figure 3:**
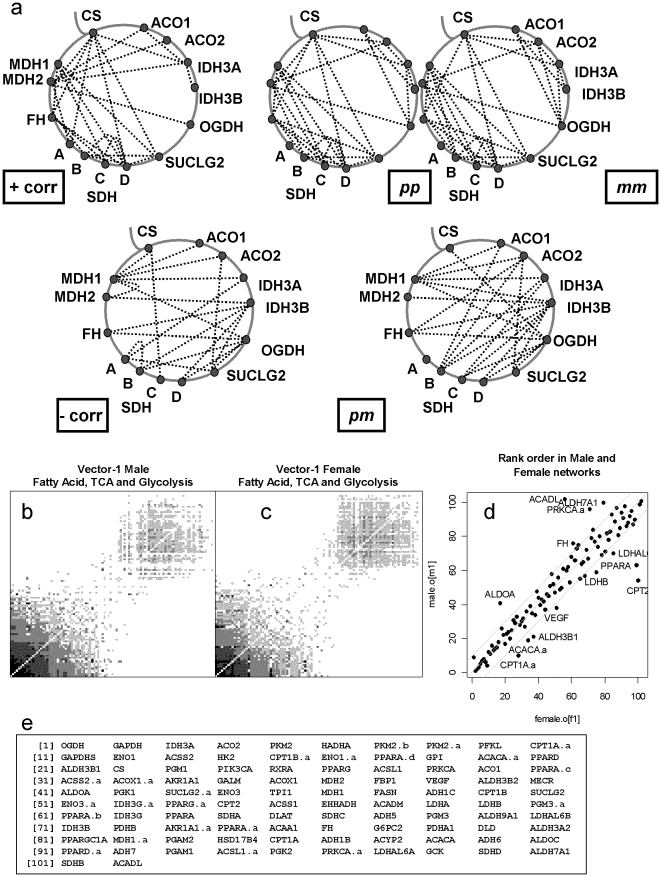
Co-expression networks for fatty acid, tri-carboxylic acid cycle, glycolysis and related genes in peripheral blood cells. The patterns of co-regulation of TCA-cycle genes by correlation and discretization are summarised (a). The correlation cut-off was set at ±0.1032, which gives approximately equal probability of accepting a gene-pair (P = 0.005) as the discretization method (quantile = 0.995). The top row shows positive co-regulation and the next row negative co-regulation. For illustrative purposes the ***pm*** graph is simplified by removing directionality from the edges. Although some of the details are different, both methods show strong co-regulation of *SDH(B,C,D)*, *FH* and *MDH1* and a weaker co-regulation of *ACO(1,2)*, *IDH3(A,B)* and *OGDH*. With both methods this second group is more clearly delineated by its negative relationships to the first group. The networks (b, c) were produced using the ***pp*** discretization method and the genes were selected using genes for three areas of metabolism using KEGG pathways [27]. Analyses were carried out, in data from GSE7965, separately for male (b) and female (c) subjects. The network was analysed using the “eigen” function from the R-package, the first eigen-vector was used to reorder the nodes. The rank of the genes from the first eigen-vector for each sex was compared (c) and over 80% of the genes lie within 10 positions of their order in the opposite sex. The genes showing the largest difference between male and female are ACADL (beta-oxidation of fatty acids), CPT2 (transport of long chain fatty acids into mitochondria), PPARA (transcription control of fatty acid and carbohydrate metabolism), CPT1A (transport of long chain fatty acids into mitochondria) and ACACA (fatty acid synthesis). (d) Comparison of gene-pairs between male and female networks, over 80% of the pairs are common. The maximum number of edges in this network is 5151 gene-pairs. The order of genes in (b) is shown in (e); the prominent cluster near the origin are genes 1:40 and the more diffuse cluster from about 55 to the end. The TCA genes in cluster 1 (*OGDH, IDH3A, ACO2*) and cluster 2 (*SDHA, SDHC, FH, SDHD, SDHB*) show that many of the relationships, found for the TCA cycle genes for both sexes, fit into a wider pattern of gene for the separate sexes.

We wished to illustrate network analysis with genes relevant to metabolic syndrome. Genes for the energy metabolic pathways glycolysis, tri-carboxylic acid cycle (TCA), fatty acid synthesis and degradation were identified from KEGG pathways [30]. A pair-list was built of all combinations of these genes. Matching pairs in our ***pp*** network for the Decode blood samples [25] were identified and these were formatted in an adjacency matrix. The TCA cycle is central to energy metabolism and here we were surprised to find that its genes are not uniformly transcriptionally regulated (**Figure 3a**). Our initial observation was made only with the genes coding for TCA cycle proteins, but we extended the analysis to include other energy pathways to find if the patterns observed with the TCA cycle fitted into a more extensive scheme. Separate analyses were performed on male and female data and about 80% (**Figure 3d**) of the gene-pairs were found in both. The networks were reordered using spectral analysis [16], using the R-function “eigen”, and similar patterns found for both sexes (**Figure 3a,b**). These gene-orderings were compared (**Figure 3c**) and found to be very similar in male and female, however some crucial genes seem to show differences. These pathways are central to metabolic control and the patterns we observe in two independent data-sets (male and female) reflect this.

## Discussion

A central aim of our approach is to identify positive and negative interactions, achieved by a discretized data-file (−1,0,1), which can be used for further analyses. We have used discretized data-files to combine data from a large study of oral cancer [15], gathered by two Affymetrix chip types (133Plus2 and 133a/b); other attempts to normalize and combine the data failed to overcome the differences between the two chip types. This result encouraged us to examine network analysis from the same starting point. An essential part of bio-discovery is to be able to map gene-clusters onto samples where they are over or under expressed. The discretized gene-sample data can be reordered using spectral analysis [16], but if a gene-cluster really behaves co-ordinately the samples can be grouped simply by selecting these genes from this file and summing the columns, to reveal the mean number of genes “off” or “on” in every sample. This ability to change the level of analysis is crucial to begin to understand possible biological associations with the patterns observed in the networks. Simple statistical tests, like Chi-square, can be used to evaluate defined gene patterns (on- or off-together, or off-on) with sets of single nucleotide polymorphisms (SNPs) or with sex or some disease or lifestyle factor.

The use of SynTReN simulated data allows us to examine the mapping between the *E coli* relationships, used to build the data files, and our determined relationships. The most simplistic comparison is the comparison between our network pair-lists and the *E coli* definitions; as we have two classes of networks (***pp*** and ***mm***) and ***pm***, we expect a difference in the types of relationship found. These comparisons are shown in **Table 1**.

Both ***pp*** and ***mm*** networks mainly identify the positive ***ac*** definitions while ***pm*** identifies mostly the repressive ***re***. More predicted pairs, defined by transitive relationships from the E coli gene-interactions, give a much better match to our calculated networks. Networks based on correlation analysis allow us to select gene-pairs which are significant by both methods, discarding many FALSE relationships from SynTReN data, but reveal some highly reproducible FALSE pairings, presumably due to consistent generation of “random” numbers. This makes SynTReN unsuitable to evaluate the use of multiple sampling to discard non-significant gene-pairs.

A systems biology approach suggests that many compensatory changes in gene-expression will be found in random samples of any human population. Some variability is due to genetic [6] or environmental [13], including dietary effects. Here we do not explore the cause of variability but test the idea that if compensatory changes occur regularly they should lead to many more correlations between genes being observed than would occur by chance. We chose two methods of detecting positive and negative co-expression – correlation and discretization, with co-occurrence assessed by Monte Carlo (MC) sampling. The two approaches agree, finding millions of gene-pairs in common and few positive co-expression relationships matching negative co-expression by the other method. The consistency of the detected patterns was further confirmed by the same patterns being frequently identified in two randomly-selected samples (620 subjects in each) from the SAFHS; multiple sampling of this dataset results in a convergence to a core set of gene-pairs which are present in all independent runs, these form about 50% of the network discovered by a single analysis, but comparison of 2 sets of shared pairs, each from 2 random samplings, showed about 80% shared pairs. The relationships capable of being described by our discretization method add biologically relevant information not available by correlation analysis. Two genes may be up-regulated together under the control of one transcriptional factor, but in the absence of that factor they might be independently controlled; if that were true our expectation would link the genes only in the “up-together” (***pp***) network. Considerations of the presence or lack of symmetry therefore add to our analytical toolbox. With correlation analysis two genes have three possible relationships – positive or negative correlation or no significant link.

Biological consistency was explored by comparing two independent studies of peripheral white-blood cell derived samples. Despite the differences in generating the data, along with the microarrays being carried out on different platforms, hundreds of thousands of gene-pairs were found in common in the two data-sets.

The discretization method was examined by the size (number of edges) of a co-expression network from actual data and from a randomly shuffled discretized matrix (**Figure 3b**). The shuffled matrix reveals the number of gene-pairs that are likely to be due to chance, given the true variability of each gene. The discovered networks were over 20-fold larger and shown to have a significance of P approximately zero, by t-test. All these criteria demonstrate that co-regulation is observable and is at least partly revealed in our networks. We demonstrate that the genes of central metabolic pathways can be used to interrogate the co-expression networks and to reveal previously unreported details. Spectral analysis reveals a clear division of this network into 2 sets of nodes and the genes which show the biggest difference in the networks for males and females contain some plausible pivot genes for metabolic control (*PPARA, CPT1A, CPT2, PRKCA and ACACA*). Despite these differences between the male and female networks the similarities are significant, about 80% of the edges are shared. From the bio-discovery viewpoint it is important to take a set of genes of known relevance and to find out how they are controlled in a large observational study, then to be able to analyze observed patterns and find out how they are affected by known biometrics or treatment regimes.

We speculate that these networks, which we have shown to contain many more edges than would occur by chance, may represent patterns of co-regulation which may include possible molecular regulatory partners without implying that there are direct causal links, however we are aware that in at least some cases the molecular interaction argument is not correct. The Cheung and Spielman data are likely to be free of heterogeneity of growth and nutrition but the immortalization procedure carries a risk of fixing differences at the time of establishing the cell-lines. Detecting common patterns of transcription between the two datasets is a strong indication that some of the patterns we observe are conserved despite environmental and other differences.

Generations of biochemists have viewed the TCA cycle almost as dogma; here we show a clear difference in two subsets of the genes on the two sides of the cycle schema. SDH(B,C,D), FH and MDH1 appear to be strongly co-regulated and are negatively co-regulated with ACO(1,2), IDH3(A,B) and OGDH; which in turn are more weakly co-regulated. This is consistent with the many other metabolic roles these enzymes play apart from their place in the TCA cycle. The ability to focus on a set of genes with an apparently well-understood role is an important aspect of being able to easily dissect and focus on small parts of an otherwise humanly unknowable network.

Regulatory links may be revealed by our networks, but biological experimentation is essential to confirm this, so our networks provide detailed information in a well-ordered manner, allowing a rational design of perturbation experiments. The second advantage of a network approach is to rapidly gain an overview of the patterns of expression relevant to any biologically defined process. Here, by simply defining the genes involved in energy metabolism, we were able to find co-expression patterns in white-blood cell derived samples – the biological drivers for the patterns are then open to investigation. Using such patterns together with the discretized gene-sample matrix it is simple to look for association between a set of genes being switched on with patient biometrics or treatment.

### Conclusions

Discretization with our co-expression analyses successfully identifies most of the defined relationships used to construct the SynTReN synthetic “microarray data”. It also detects many transitive relationships which are constrained to exist by the presence of common activators or repressors. The co-expression analysis, compared with correlation analysis, identifies many shared gene-gene relationships in observational microarrays, even when the platform for carrying out the mRNA analysis is different. The co-expression of metabolic-related genes in males and females is shown to be largely similar, but find a number of differences in known control genes. The results indicate that the described method can be used to identify real relationships, suggesting that the discretized data is a useful adjunct to reveal patterns in gene-expression data.

## Methods

Genes with unexpectedly high or low values, compared to their mean values, are classed as 1 or −1 respectively, using the method of Quackenbush [31], where each sample is compared to the mean of all samples in the dataset. This discretized matrix is used to derive two matrices, **P** and **M**, holding the positive and negative information in all positive forms. The transpose of these matrices (**P'** and **M'**) are then used to calculate the inner-products, **P.P'**, **M.M'** and **P.M'**; these matrices record the sum of all samples in which each gene-pair is recorded. The scores are evaluated against a calculated expectation (P = 0.005), by Monte Carlo sampling [32]. The inner-products (**P.P'**, **M.M' and P.M'**) are adjacency matrices and record the number of samples in which the accepted gene-pairs are found. For computational purposes, the adjacency matrices for **P.P'** and **M.M'** are stored in the upper-triangular form with each gene-pair represented only once and diagonal entries are set to zero. **P.M'** stores the up-down relationships and is asymmetric. To represent the relationships identified by the discretization method we use the following terminology: genes ***mm*** (minus:minus, down-together), ***pp*** (plus:plus, up-together) and ***pm*** (plus:minus, up-down). The adjacency matrices are converted into pair-lists: ***pp***, ***mm*** and ***pm*** which are used to compare networks from different datasets; in the case of ***pm*** gene1 is up and gene2 is down.

We wished to filter out relationships that were likely to be due to chance, given the density, or number of 1's for each gene. Using Monte Carlo sampling methods [32] we estimated the distribution of scores for randomized vectors of all possible densities, by permuting the order of each and then recording the number of times 1's occur for both vectors at each position. The test was repeated 1000 times for every pair of vectors and the values which exceeded 99.5% of the random scores (calculated by the R-package [33] function *quantile* [34]) were accepted. We estimated the false positive rate by randomizing the order of each gene vector in the discretized gene-sample matrix, then constructing the matrices – this gives around 5% of the edges found with un-shuffled data.

To examine detailed information, the matrices were converted to edges (gene-pairs) including the number of samples where the relationship was found (**Figure 2**). Two graphs can be compared to find the number of edges in common (*intersection*). In the ***pp*** or ***mm*** graphs the order of the nodes is not significant, but in the ***pm*** graph we use the convention where node-1 of each pair is ***p*** and node-2 is ***m***. The ordered ***pm*** structure allows evaluation of pairs for both ***p→m*** and ***m←p*** relationships; so the ***pm*** matrices are square and asymmetric with directed edges.

Lower Z-score cut-offs give better detection sensitivity to the *E coli* definitions (**Table 9**), but the lowest value we used was Z = 0.4, as we want to build our networks using observable changes in gene-expression.

**Table 9:** Effect of changing Z-score on Analysed Network Estimation.

		*ac*	*du*	*re*
Z = 0.4	*pp* *mm* *pm*	80890	454	2329
Z = 0.8	*pp* *mm* *pm*	71710	335	1226
Z = 1.2	*pp* *mm* *pm*	35410	124	0015
Z = 1.4	*pp* *mm* *pm*	14160	001	009
Z = 1.6	*pp* *mm* *pm*	13140	001	0010

The SynTReN simulated data for 100 samples was analysed using different Z-scores to select up- and down-regulated genes. Although the specificity increased at higher Z-scores the sensitivity was lower. Our strategy in looking for bio-markers is to accept relationships with lower significance at this stage but subsequently require that any useful pattern or clique is highly connected. In real situations, it is also important to require that the cliques are found in independent datasets. Our decision not to look at lower Z-scores than 0.4 is based on pragmatic biomarker requirements, where changes in expression have to be robust and indicate changes likely to be found by other methods.

The analysis described was carried out on three datasets: GSE5859 immortalized lymphoblastoid cells [24] referred to here as the “Cheung and Spielman” data, downloaded from the Gene Expression Omnibus repository (http://www.ncbi.nih.gov/geo), the San Antonio Family Heart Study (SAFHS), TABM305 [23], downloaded from ArrayExpress (http://www.ebi.ac.uk/microarray-as/ae/) and the Decode study, GSE7965 [25], from GEO; these were chosen as they represented large independent studies derived from white blood cells. The SAFHS was very large (1239 samples used here) and enabled random subdivision (groups of 620 and 619) to compare independent sets of samples from the same source.

R-scripts and perl programs, to carry out the analyses described, are available on-line (http://sourceforge.net/projects/gene-expression/).

We have compared networks from discretization and correlation analysis and have tried to use approximately equal probability cut-offs for both methods, with P approximately 0.005; for discretization this is set by the co-occurrence score distribution from the Monte Carlo sampling, and correlation by t-test using the formula: 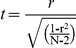 where ***r*** is the Pearson correlation coefficient, and **N** is the number of observations.
